# Case report: Partial cystectomy for pheochromocytoma of the urinary bladder: A case report and review of literature

**DOI:** 10.3389/fcvm.2022.1092260

**Published:** 2022-12-19

**Authors:** Liang-Liang Hu, Zhong-Qiang Guo, Peng Dai, Gang Chen, Tao Tian

**Affiliations:** ^1^Department of Urology, Shandong Zaozhuang Municipal Hospital, Zaozhuang, China; ^2^Department of Urology, People's Hospital of Yicheng District, Zaozhuang, Shandong, China; ^3^Department of Urology, Jinan Seventh People's Hospital, Jinan, Shandong, China

**Keywords:** bladder neoplasms, pheochromocytomas, partial cystectomy, hypertension, case report

## Abstract

Pheochromocytomas are neuroendocrine tumors that produce catecholamines and can be difficult to diagnose. Bladder involvement is uncommon with pheochromocytoma. Hypertension (sometimes with hypertensive crisis coinciding with micturition), headache, hematuria and syncope, which are commonly associated with voiding, are the most prevalent symptoms. While transurethral resection may be performed in roughly 20% of patients, 70% require partial cystectomy and 10% require radical cystectomy. We present a case of pheochromocytoma with hypertension and syncope that was often associated with voiding, satisfactorily treated by partial cystectomy.

## Introduction

The neuroendocrine tumor pheochromocytoma secretes and metabolizes catecholamines. Due to the excessive release of catecholamines, pheochromocytoma is associated with a variety of clinical symptoms, including hypertension, headache, palpitations, perspiration, and other cardiovascular signs. The metabolic action of catecholamines is reflected in various symptoms of pheochromocytoma (1). Based on the World Health Organization classification (2004), pheochromocytoma is categorized as intrarenal and extrarenal, with 80–85% of the tumors occurring in the adrenal medulla and 15–20% in extrarenal sites (2). The bifurcation of the aorta or the inferior mesenteric artery is the most common site for extrarenal pheochromocytoma (3). Surgery is the most common treatment for pheochromocytoma; however, abrupt fluctuations in intraoperative hormone levels cause changes in blood pressure, chronic hypotension and other symptoms, thereby complicating the treatment and increasing risk of mortality/complications (4). To our knowledge, there are limited reports of ectopic pheochromocytoma of the bladder (5). In the present study, we report an uncommon case of ectopic pheochromocytoma of the bladder with palpitations during micturition.

## Case report

**Figure 1:**
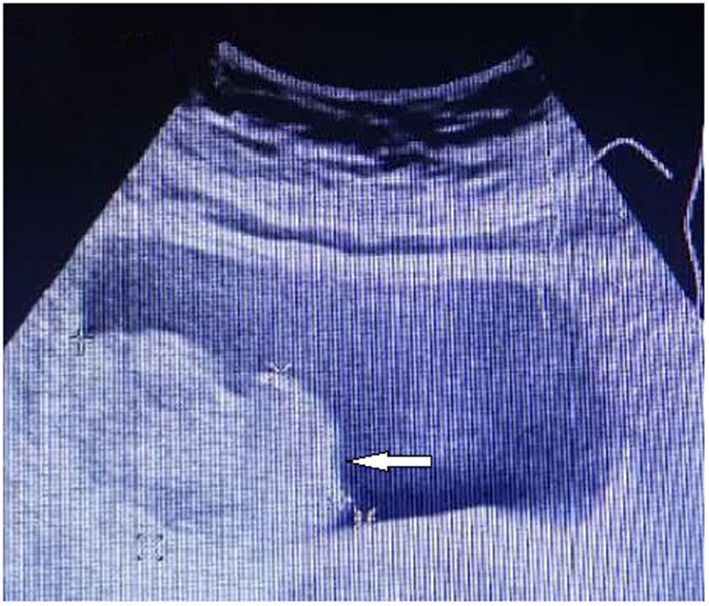
Color Doppler showing the lesion in the right wall of the urinary bladder.

**Figure 2:**
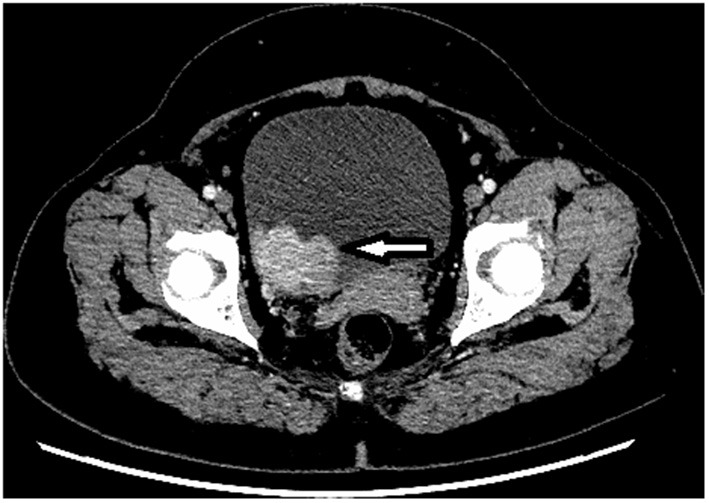
CT enhancement showing the lesion in the right wall of the urinary bladder.

**Figure 3:**
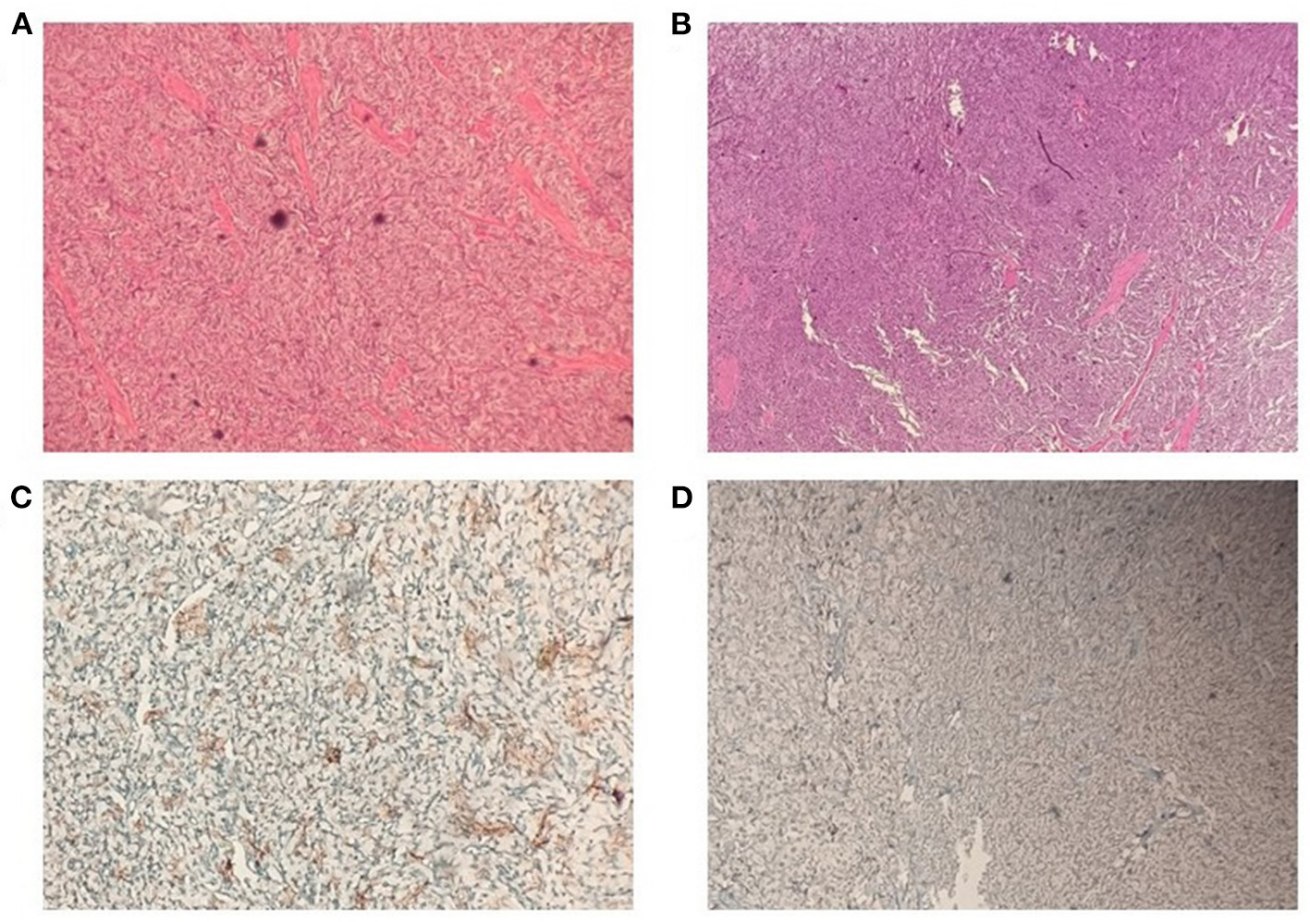
Microscopic examination of pheochromocytoma. **(A,B)** Haematoxylin and eosin staining. **(C)** Immunohistochemical staining of tumor cells was positive for chromogranin A. **(D)** Immunohistochemical staining of tumor cells was positive for synaptophysin.

A 52-year-old Asian woman was admitted to the Department of Cardiology, Zaozhuang Municipal Hospital on April 18th, 2019 due to paroxysmal palpitations. The patient first experienced palpitations and weariness before 4 months, which were frequently followed by exhaustion, emotional agitation and urination. Occasionally, the patient also had symptoms such as headache, nausea and vomiting, which were partially relieved by rest. She had a history of atrial fibrillation and was prescribed rivaroxaban 20 mg three times a day regularly. The patient did not have any harmful habits, such as smoking or alcohol consumption. She got married at the age of 26 years and has been menopausal since 2018. Her husband and children were healthy, and there was no family history of genetic diseases. Color Doppler ultrasonography was performed, and the right posterior wall of the bladder was probed, which revealed a solid isoechoic mass measuring ~5.4 × 4.0 × 2.6 cm, with abundant blood flow (Figure 1). CT enhancement revealed that the cross-section of the right posterior wall of the bladder appeared as a soft tissue shadow measuring ~5.0 × 3.7 cm, with an irregular form and a wide base (Figure 2). The bladder displayed clear enhancement after stimulation, and both kidneys were free of hydronephrosis. A 24-h urine study revealed normal levels of norepinephrine, epinephrine and vanillylmandelic acid (VMA). In preparation for tumor resection, the patient was treated with alpha blockade and adequate hydration for 10 days. A solid mass ~5 cm in diameter was discovered during the procedure on the right side of the bladder. It was adjacent to the right ureter and had a smooth surface. The decision was made to perform a partial cystectomy. Intraoperative blood pressure ranged from 41 to 157/61 to 280 mmHg, with a heart rate of 42–153 beats per minute. Bladder pheochromocytoma was confirmed *via* pathological assessment after the surgery (Figure 3). The palpitations and weariness associated with micturition decreased after the procedure, and there were no signs of hormone deficiency. The patient underwent cystoscopy every 6 months after surgery with no signs of recurrence.

## Discussion and review of the literature

Pheochromocytoma is a neuroendocrine tumor originating from pheochromocytes that is most seen in the paraganglion cells or the adrenal medulla and adrenal medulla, with an incidence of 0.1–0.3% in persons with hypertension (6). Paroxysmal hypertension, palpitations, tachycardia, headache, anxiety and tension are the frequent clinical signs of pheochromocytoma (7). Clinicians who suspect the tumor should look for a variety of symptoms and clinical presentations. Pheochromocytoma is characterized by increased catecholamine production (8). In the present study, laboratory investigations revealed a high adrenaline level, which is a sign of pheochromocytoma. In addition, palpitations were common after micturition in our patient, which is consistent with catecholamine excess. The majority of pheochromocytomas are identified on CT scans or B-ultrasound (9). Extrarenal pheochromocytomas are uncommon in adults; however, they account for 30–40% of all pheochromocytomas in children (10). Urinary bladder pheochromocytoma occurs in <1% of the extrarenal cases and accounts for 0.06–0.33% of bladder tumors. It arises from paraganglion cells in the bladder wall, is more common in the posterior wall, and is least common in the trigone. Women are more commonly affected than men (11). In clinical practice, the occurrence of pheochromocytoma of the bladder is low, especially in individuals with unusual clinical symptoms; therefore, it is more likely to be ignored or misdiagnosed, which increases the need of surgery.

We statistically counted cases of pheochromocytoma of the bladder reported in PubMed over the last 8 years and evaluated meaningful variables including region, gender, age, maximum blood pressure levels, urinary catecholamine levels, tumor size, histological examination results, and treatment modality (Supplementary Table 1). The majority of pheochromocytomas are benign, with just 2–13% becoming cancerous. Some studies have reported that roughly 10% of ectopic pheochromocytomas are malignant (12). Ectopic pheochromocytoma has been reported to be a locally aggressive vascular tumor that likely spreads to lymph nodes (13). Most patients have a fair prognosis with proper diagnosis and therapy. Pheochromocytoma is treated by the surgical excision of the tumor (14). Transurethral resection of bladder tumor (TURBT), partial cystectomy, radical complete cystectomy and other procedures are performed for the treatment of bladder pheochromocytoma. A partial cystectomy was the best option for the patient in this study.

Considering that a significant quantity of catecholamines is released by the tumor, regular preoperative preparation, expansion, and hypotension are suggested for all patients with pheochromocytoma of the bladder. The use of alpha-blockers is the most important aspect to improve surgical safety and lower mortality in patients with pheochromocytoma [16]. In addition, proper preoperative therapy can help to avoid intraoperative and postoperative problems caused by a hypertensive crisis. In the present case, although the patient's blood pressure was normal at the time of admission, we prescribed prazosin 1 mg orally thrice a day for 10 days before surgery to prevent intraoperative blood pressure variations.

Finally, there are a few takeaways from this study. First, urologists should be aware that ectopic pheochromocytoma might occur in the bladder, which is an uncommon but probable location. Therefore, pelvic imaging, such as a CT scan or B-ultrasound, should be part of a standard screening program for pheochromocytoma. Second, surgery is the best therapeutic option for pheochromocytoma. TURBT, partial cystectomy, radical complete cystectomy and other procedures can be performed to treat bladder pheochromocytoma. Finally, regular preoperative preparation for bladder pheochromocytoma, including volume expansion and hypotension, as well as alpha blockade, should be done before the surgery. A cystoscopy with biopsy is not indicated to avoid an adrenal crisis.

## Data availability statement

The original contributions presented in the study are included in the article/Supplementary material, further inquiries can be directed to the corresponding author/s.

## Ethics statement

Ethical review and approval was not required for the study on human participants in accordance with the local legislation and institutional requirements. Written informed consent for participation was not required for this study in accordance with the national legislation and the institutional requirements.

## Author contributions

L-LH wrote this article. PD and Z-QG collected data for this article. TT helped draft the manuscript and gave final approval of the version to be published. All authors read and approved the final manuscript.
