# Supplementary material for: Case report: Partial cystectomy for pheochromocytoma of the urinary bladder: A case report and review of literature
Source: Front Cardiovasc Med. 2022 Dec 19;9:1092260. doi: 10.3389/fcvm.2022.1092260 (PMC9806206; doi:10.3389/fcvm.2022.1092260)
Supplement: Supplementary file 1 [file Table_1.DOCX]

| **Supplementary Table 1. Case reports of pheochromocytoma of the urinary bladder from 2015 to 2022** | | | | | | | | | |
| --- | --- | --- | --- | --- | --- | --- | --- | --- | --- |
| List | Author, published year | Country | Age | Sex | Maximum blood pressure | Catecholamine levels in urine | Dimension of the tumor | Histological examination results | Treatment modality |
| 1 | Kroiss, A.S, et al.2018 | Austria | 40 | male | NA | Increased | NA | PHEO | partial cystectomy |
| 2 | Hu, W, et al .2017 | China | 55 | female | NA | Normal | 6.2×5.9 cm | Composite PHEO | Surgical resection |
| 3 | Ching, D.E. 2016 | Austria | 77 | female | NA | NA | NA | paraganglioma | Surgical resection |
| 4 | Chen Y.W et al. 2016 | China | 52 | female | 190/110 | Increased | 1.7 ×2.4 cm | PHEO | TURBT |
| 5 | Bishnoi, K, et al. 2016 | India | 55 | male | 190/100 | Normal | 4.1 × 5.8 cm | paraganglioma | partial cystectomy |
| 6 | Valsangkar, R.S, et al. 2015 | India | 21 | male | 230/110 | Normal | 2.6 cm | paraganglioma | partial cystectomy |
| 7 | Quist, E.E, et al. 2014 | USA | 58 | male | 300 mmHg | Normal | 8 cm | paraganglioma | radical cystoectomy |
| 8 | Sharma A, et al. 2018 | India | 40 | male | NA | Increased | 3.8-4.3 cm | PHEO | TURBT |
| 9 | Roberto S, et al. 2015 | USA | 66 | male | 200 mmHg | Increased | 2 cm | PHEO | TURBT |
| 10 | Poonam S, et al. 2015 | India | 45 | female | NA | Increased | 2×2 cm | paraganglioma | Surgical resection |
| 11 | Peng, C, et al. 2014 | China | 61 | male | 168/100mmHg | NA | 3.9x3.6 cm | paraganglioma | partial cystectomy |
| 12 | Katiyar, R, et al. 2015 | India | 32 | female | 120/78 mmHg | NA | 2.6×2.4 cm | paraganglioma | TURBT |
| 13 | Katiyar, R, et al. 2015 | India | 45 | female | 118/84mmHg | NA | 2.4×2.6 cm | paraganglioma | TURBT |
| 14 | Iwamoto, G, et al. 2017 | Japan | 77 | male | 240 mmHg | Increased | 2.6 cm | paraganglioma | TURBT |
| 15 | Chaaya, G, et al. 2018 | Hispanic | 28 | female | NA | Increased | 4.6 cm | paraganglioma | TURBT |
| 16 | Priyadarshi, V. and D.K. Pal. 2015 | India | 58 | female | 236/118mmHg | NA | 1.5×1.0 cm | paraganglioma | TURBT |
| 17 | Lazareth, H, et al. 2017 | France | 67 | female | 240/110mmHg | Normal | 1.6×1.6 cm | PHEO | TURBT |
| 18 | Williams, P, L. Siref, et al. 2017 | USA | 57 | male | 171/88 | Increased | 2.1x1.3 cm | PHEO | partial cystectomy |
| 19 | Maric, P, et al. 2016 | Serbia | 49 | female | 220/130mmHg | Normal | 3.2 × 2.8 cm | PHEO | partial cystectomy |
| 20 | Nerli, R.B, et al. 2015 | India | 18 | male | 200/110mmHg | NA | 6×5 cm | PHEO | partial cystectomy |
| 21 | Kido, K, et al. 2018 | Japan | 59 | male | 290 mmHg | Increased | 3.8 cm | paraganglioma | TURBT |
| 22 | Youssef, A. and A. Hamade. 2017 | UK | 57 | female | NA | Normal | NA | PHEO | TURBT |
| 23 | Han, Y.J, et al. 2015 | Korea | 61 | female | 230/110mmHg | Increased | 3×3.2 cm | PHEO | partial cystectomy |
| 24 | Gkikas, C., M. Ram, and P. Tsafrakidis, 2016 | UK | 66 | male | NA | NA | 5 cm | paraganglioma | TURBT |
| 25 | You, D, et al. 2016 | China | 23 | male | 160̸100 mmHg | Increased | NA | PHEO | radiotherapy |
| 26 | Ejaz, S, et al.2022 | DRC | 43 | female | 154/86 mmHg | Increased | 2 cm | paraganglioma | resection of bladder |
| 27 | Yoo, K.H, et al.et al. 2020 | USA | 53 | male | NA | NA | 3 cm | paraganglioma | Surgical resection |
| 28 | Choi, Y.H. and D.S. Lee. 2020 | Korea | 76 | male | NA | NA | 2.0x1.7cm | paraganglioma | partial cystectomy |
| 29 | Hafiz, B., et al. 2021 | EGY | 36 | female | NA | NA | 8.2 x 6.4 cm | PHEO | partial cystectomy |
| 30 | Urabe, F, et al. 2019 | Japan | 64 | female | 300 mmHg | Increased | 2.0 x 2.1 cm | paraganglioma | partial cystectomy |
| 31 | Ranjan, R, et al. 2019 | India | 55 | female | NA | Increased | 4 x 4 cm | paraganglioma. | partial cystectomy |
| 32 | Emuze, M.E. 2022 | Nigeria | 32 | male | 140/104mmHg | NA | NA | NA | Refusing surgery |
| 33 | Sugimura, R, et al. 2019 | Japan | 56 | female | 230mmHg | Normal | NA | paraganglioma. | partial cystectomy |
| 34 | Teragaki, M, et al. 2020 | Japan | 46 | female | 156/112mmHg | Increased | 15×11 cm | paraganglioma. | partial cystectomy |
| 35 | Johnson, J.T.et al. 2020 | India | 44 | female | 126/80mmHg | Increased | 2×1 cm | paraganglioma | Surgical resection |
| 36 | Wen, C.Y, et al. 2021 | China | 32 | female | NA | NA | 4.5 cm | paraganglioma | partial cystectomy |
| 37 | Parra, L.A, et al. 2020 | USA | 61 | male | NA | NA | NA | paraganglioma | TURBT |
| 38 | Chen, J. and H.F. 2022 | China | 22 | female | 110/68 mmHg | Normal | 2.5× 2.1 cm | paraganglioma | partial cystectomy |
| 39 | Kurosawa, S. et al. 2022 | Japan | 33 | female | 223/162mmHg | NA | NA | PHEO | biopsy |
| 40 | Chan, V.S.H, et al. 2019 | China | 6 | female | NA | Increased | 6 cm | PHEO | surgical resection |
| 41 | Muhammad, S, et al. 2021 | PAK | 13 | female | 150/90mmHg | NA | NA | paraganglioma | surgical resection |
| 42 | Rajkumar, B, et al. 2022 | IND | 55 | female | NA | NA | 4.1x3.4 cm | PHEO | TURBT |
| 43 | Matsuzawa, N, et al. 2022 | Japan | 64 | male | 121/67 mmHg | NA | 7.7 cm | paraganglioma | radical cystoectomy |
| 44 | Sonmez, G, et al. 2020 | Turkey | 23 | female | NA | NA | 34 x 33 cm | paraganglioma | TURBT |
| 45 | Jandou, I, et al,2020 | Morocco | 39 | female | 22/140 mmHg | Increased | 5.3 × 5.0 × 6.0 cm | PHEO | partial cystectomy |
| 46 | Alkhatatbeh, H, 2020 | Jordan | 62 | male | 220/120mmHg | NA | 4.3× 3.5 cm | PHEO | TURBT |
| 47 | Zhou, J. et al. 2020 | China | 44 | male | 126/78 mm Hg | Increased | 2.2 cm | PHEO | TURBT |

[1-46]NA:Not Applicable

PHEO: Pheochromocytoma

TURBT: Transurethral Resection of Bladder Tumor

**Reference**

1. Bishnoi, K., et al., Bladder paraganglioma: safe and feasible management with robot assisted surgery. J Robot Surg, 2016. 10(3): p. 275-8.

2. Chaaya, G., et al., Paraganglioma of the Urinary Bladder: A Rare Cause of Hypertension and Urinary Tract Infections. Am J Med Sci, 2018. 355(2): p. 191-194.

3. Ching, D., E. Anastasiadis, and S. Sandhu, An unusual case of asymptomatic non-urothelial bladder tumour. Int J Surg Case Rep, 2016. 27: p. 18-20.

4. Gkikas, C., M. Ram, and P. Tsafrakidis, Urinary Bladder Paraganglioma and Concomitant Metastatic Lung Cancer. A Case Report. Urol Case Rep, 2016. 5: p. 17-9.

5. Han, Y.J., et al., Thunderclap-like headache triggered by micturition and angina as an initial manifestation of bladder pheochromocytoma. A case report. Sao Paulo Med J, 2015. 133(2): p. 154-9.

6. Hu, W., et al., A rare and easily misdiagnosed tumor of the urinary bladder: primary composite pheochromocytoma. Int J Clin Exp Pathol, 2017. 10(10): p. 10522-10530.

7. Iwamoto, G., et al., Paraganglioma in the bladder: a case report. J Med Case Rep, 2017. 11(1): p. 306.

8. Katiyar, R., et al., Non-Functional Paraganglioma of the Urinary Bladder Treated by Transurethral Resection: Report of Two Cases. J Clin Diagn Res, 2016. 10(2): p. XD01-XD03.

9. Kido, K., et al., Partial Cystectomy of Paraganglioma of the Urinary Bladder Before Living Kidney Transplantation: Case Report. Transplant Proc, 2018. 50(3): p. 898-901.

10. Kroiss, A.S., et al., A rare case of a 123I-MIBG SPECT/CT positive, but 68Ga-DOTA-TOC PET/CT negative pheochromocytoma of the bladder. Revista Española de Medicina Nuclear e Imagen Molecular (English Edition), 2018. 37(5): p. 315-317.

11. Lazareth, H., et al., Paraganglioma of the bladder in a kidney transplant recipient: A case report. Mol Clin Oncol, 2017. 6(4): p. 553-555.

12. Maric, P., et al., Pheochromocytoma of the urinary bladder--A case report. Vojnosanit Pregl, 2016. 73(6): p. 584-7.

13. Nerli, R.B., et al., Pheochromocytoma of the Urinary Bladder - A Case Report of an Unusual Presentation. Indian J Surg Oncol, 2015. 6(3): p. 303-6.

14. Peng, C., et al., Non-functioning paraganglioma occurring in the urinary bladder: A case report and review of the literature. Oncol Lett, 2015. 10(1): p. 321-324.

15. Priyadarshi, V. and D.K. Pal, Paraganglioma of urinary bladder. Urol Ann, 2015. 7(3): p. 402-4.

16. Quist, E.E., et al., Malignant paraganglioma of the bladder: a case report and review of the literature. Pathol Res Pract, 2015. 211(2): p. 183-8.

17. Salvatori, R., et al., Bladder pheochromocytoma. Endocrine, 2015. 48(1): p. 349-50.

18. Sharma, A.P., et al., Management of bladder pheochromocytoma by transurethral resection. Asian Journal of Urology, 2019. 6(3): p. 298-301.

19. Sherwani, P., et al., Concurrent nonfunctional paraganglioma of the retroperitoneum and urinary bladder: A case report with literature review. Indian J Radiol Imaging, 2015. 25(2): p. 198-201.

20. Valsangkar, R.S., et al., Bladder paraganglioma with renal agenesis: A possible new association and its implications in the light of REarranged in transfection gene genetics. Urol Ann, 2015. 7(3): p. 410-3.

21. Wen, C.Y., C.T. Yu, and C.H. Hsieh, Atypical presentation of bladder pheochromocytoma. Ci Ji Yi Xue Za Zhi, 2017. 29(1): p. 46-49.

22. Williams, P., L. Siref, and M. Feloney, Pheochromocytoma of the bladder. JAAPA, 2017. 30(10): p. 23-25.

23. You, D., et al., Radiotherapy for urinary bladder pheochromocytoma with invasion of the prostate: A case report and literature review. Mol Clin Oncol, 2016. 4(6): p. 1060-1062.

24. Youssef, A. and A. Hamade, Pheochromocytoma: A Cause of Anemia. Urol Case Rep, 2017. 11: p. 53-54.

25. Alkhatatbeh, H., et al., Urinary bladder pheochromocytoma managed with TURBT. Case report and review of literature. Urol Case Rep, 2020. 33: p. 101291.

26. Chan, V.S.H., et al., Occult extra-adrenal pheochromocytoma in the urinary bladder. BMJ Case Rep, 2019. 12(3).

27. Chen, J. and H.F. Yang, Nonfunctional bladder paraganglioma misdiagnosed as hemangioma: A case report. World J Clin Cases, 2022. 10(15): p. 4929-4934.

28. Choi, Y.H. and D.S. Lee, Bladder Paraganglioma Mimicking a Tumor Contained in a Ureterocele. Urology, 2020. 142: p. e36-e38.

29. Ejaz, S., et al., A Study of Paraganglioma Cases With Non-European Ancestry. Cureus, 2022. 14(8): p. e27854.

30. Emuze, M.E., et al., Extra-adrenal phaeochromocytoma in a resource poor setting: A case report. Endocr Regul, 2022. 56(1): p. 48-54.

31. Hafiz, B., et al., Concurrent Urinary Bladder Paraganglioma and Adrenal Phaeochromocytoma With Succinate Dehydrogenase-B Mutation. Cureus, 2021. 13(8): p. e17350.

32. Jandou, I., et al., The ectopic vesical pheochromocytoma a diagnostic and therapeutic challenge case report and literature review. Int J Surg Case Rep, 2020. 77: p. 857-861.

33. Johnson, J.T., et al., Micturition syncope secondary to urinary bladder paraganglioma. BMJ Case Rep, 2020. 13(3).

34. Kurosawa, S., et al., Major intraoperative bleeding and drastic change in circulatory dynamics in a pregnant patient with metastatic pheochromocytoma: a case report. JA Clin Rep, 2022. 8(1): p. 13.

35. Matsuzawa, N., et al., Paraganglioma of the urinary bladder initially diagnosed as gastrointestinal stromal tumor requiring combined resection of the rectum: a case report. World J Surg Oncol, 2022. 20(1): p. 185.

36. Muhammad, S., et al., Paraganglioma of Urinary Bladder in a Pediatric Patient. Cureus, 2021. 13(3): p. e13964.

37. Parra, L.A., et al., Multifocal Urinary Bladder Paragangliomas With Negative 68Ga-DOTATATE Uptake and Positive 123I-MIBG Uptake. Clin Nucl Med, 2020. 45(3): p. e156-e157.

38. Rajkumar, B., et al., Primary Neuroendocrine Tumor of Urinary Bladder: A Case Report of an Unusual Occurrence. Cureus, 2022. 14(2): p. e22720.

39. Ranjan, R., et al., Extending Horizon of Robotic Surgery to Bladder-Preserving Approach for Vesical Paraganglioma: Rare Case with Unusual Presentation. J Endourol Case Rep, 2020. 6(4): p. 319-321.

40. Sonmez, G., et al., Symptomatic paraganglioma of the urinary bladder: A rare case treated with a combined surgical approach. Urol Case Rep, 2020. 33: p. 101290.

41. Sugimura, R., et al., Functional paraganglioma of the bladder: Both radiographic-negative and laboratory-negative case. IJU Case Rep, 2019. 2(4): p. 174-177.

42. Teragaki, M., et al., Miction-induced Hypertension Disclosed by Home Blood Pressure Measurement in a Patient with Small Paraganglioma. Intern Med, 2020. 59(6): p. 793-797.

43. Urabe, F., et al., Combination of en bloc transurethral resection with laparoscopic partial cystectomy for paraganglioma of the bladder. IJU Case Rep, 2019. 2(5): p. 283-286.

44. Wen, C.Y., et al., Modified Dual Docking Robotic Surgery for Hereditary Paraganglioma-Pheochromocytoma Syndrome. Cureus, 2021. 13(8): p. e16947.

45. Yoo, K.H., et al., Aggressive Paraganglioma of the Urinary Bladder with Local Recurrence and Pelvic Metastasis. Pathol Oncol Res, 2020. 26(4): p. 2827-2829.

46. Zhou, J., et al., Successful transurethral resection of the prostate in ectopic prostate pheochromocytoma: A case report. Medicine (Baltimore), 2020. 99(17): p. e19852.
